# Comparative Analysis of the Heptahelical Transmembrane Bundles of G Protein-Coupled Receptors

**DOI:** 10.1371/journal.pone.0035802

**Published:** 2012-04-24

**Authors:** Tetsuji Okada

**Affiliations:** Department of Life Science, Gakushuin University, Toshima-ku, Tokyo, Japan; Medical School of Hannover, United States of America

## Abstract

**Background:**

G protein-coupled receptors represent a large family of eukaryotic membrane proteins, and are involved in almost all physiological processes in humans. Recent advances in the crystallographic study of these receptors enable a detailed comparative analysis of the commonly shared heptahelical transmembrane bundle. Systematic comparison of the bundles from a variety of receptors is indispensable for understanding not only of the structural diversification optimized for the binding of respective ligands but also of the structural conservation required for the common mechanism of activation accompanying the interaction changes among the seven helices.

**Methodology/Principal Findings:**

We have examined the bundles of 94 polypeptide chains from almost all available structures of 11 receptors, which we classified into either inactivated chain or activated chain, based on the type of bound ligand. For the inactivated chains, superposition of 200 residue bundles by secondary structure matching demonstrated that the bound ligands share a laterally limited cavity in the extracellular section of the bundle. Furthermore, a distinct feature was found for helix III of bovine rhodopsin, which might have evolved to lower its activity in the presence of 11-*cis*-retinal, to a level that other receptors could hardly achieve with any currently available ligands.

**Conclusions/Significance:**

Systematic analysis described here would be valuable for understanding of the rearrangement of seven helices which depends on the ligand specificity and activation state of the receptors.

## Introduction

G protein-coupled receptor (GPCR) mediates signal transduction through the cell membrane by activating many copies of cognate heterotrimeric G protein upon binding of an agonist [1]. GPCRs form a large superfamily of heptahelical transmembrane (7TM) proteins and function universally across the eukaryotic organisms [2]. A majority of the GPCR superfamily is grouped into the class A (rhodopsin-like) family which shares a handful of amino acids in the 7TM region. Crystallographic studies have made extensive progress in solving structures for the class A GPCRs for biogenic amines, including turkey β1 and human β2 adrenergic receptors, human dopamine D3 receptor, human histamine H1 receptor, and most recently, human M2 and rat M3 muscarinic acetylcholine receptors [3], [4]. Other GPCRs of known structure are bovine and squid rhodopsins, human A2A adenosine receptor, human CXCR4 chemokine receptor and human S1P1 receptor (Sphingosine 1-phosphate receptor 1) [5].

The orthosteric ligand binding site of the class A GPCRs mainly consists of residues within the 7TM bundle. Although accumulating crystallographic evidence has proved that the arrangements of helices in the 7TM bundle of these receptors are very similar to each other, it remains unclear whether a particular receptor group has unique features in the backbone trace of the 7TM region. Therefore, the manner in which these seven helices are able to discriminate ligands among the class A GPCRs is a fundamental issue to be investigated in detail. Although the experimental structures of GPCRs have been extensively reviewed [6], [7] and it is common for some pairwise comparisons to be described when a new structure appears, there are few systematic surveys of 7TM structures.

The structures in the presence of the inactivating ligand (antagonist, inverse-agonist) have been obtained for all the receptors described above. The agonist bound and/or ligand free structures have also become available for bovine rhodopsin, β1 and β2 receptors and A2A receptor [8]. However, some of the agonist bound structures assume inactive-like conformations [9], indicating that the structural equilibrium between the inactive and active forms of a receptor is determined by complex mechanisms.

After selecting and aligning the 7TM bundles of all available crystal structures, we performed an extensive analysis of the inactivated (antagonist bound or inverse-agonist bound) forms and found that bovine rhodopsin has a distinct rigid-body arrangement of helix III that might contribute to its extremely low activity without light stimulation. The implications of the present analyses are also discussed with regard to the studies of activation mechanisms and the modeling of rhodopsin-like receptors of unknown structure.

## Results

First, we tabulated almost all the crystallographic PDB entries for bovine and squid rhodopsins, β1 and β2 receptors, A2A receptor, CXCR4 receptor, D3 receptor, H1 receptor, M2 and M3 receptors and S1P1 receptor, excluding redundant entries with lower resolution. Each polypeptide chain in an entry was considered separately, giving a total of 94 chains. Then, they were classified into two categories: an inactivated group (antagonist bound, inverse-agonist bound) and an activated group (agonist bound, ligand free), containing 62 and 32 chains, respectively. This classification is solely based on the type of ligand binding and does not necessarily mean that each chain in the activated group substantially differs with respect to the backbone structure from the inactivated group. Since current analyses mainly focus on the inactivated chains, it is important to ensure that the inactivated group does not contain any chains of agonist-bound, inactive-like structures. A complete list of the chains is shown in Table S1 of the Supporting Information, and also available at a new web site http://www.gses.jp/7tmsp/.

According to multiple sequence alignments performed at GPCRDB (http://www.gpcr.org/7tm/), each of the polypeptide chains was processed to make a 7TM bundle consisting of 200 residues, which is intermediate in size between that described for bovine rhodopsin [10] and β2 receptor [11]. Although there are no universally accepted criteria to define the 7TM bundle, the well-conserved lengths of the first and third extracellular loops and the first and second cytoplasmic loops in class A GPCRs leave little arbitrariness in the choice for the range of each helix. At this stage, 12 chains of the inactivated form and 2 chains of the activated form were found to be <200 residues in size, and were not processed further.

The range of each helix contained in a bundle is shown in the footnote of Table 1. The bundle of 200 residues chosen in this work does not exactly correspond to the region buried within the expected membrane thickness. For instance, the carboxyl termini of helices III and V, and the amino terminus of helix VI protrude past the cytoplasmic membrane surface. It is important to include these cytoplasmic regions as parts of the bundle, because they are supposed to regulate both the constitutive and ligand-induced activities of the receptors [7]–[9]. The length of helix I is currently limited by the availability of the coordinates, and the segment analyzed is probably shorter than the thickness of the membrane.

**Table 1:** Averaged rmsds (Å) of the 7TM bundle pairs in the inactive state for class A GPCRs of known structure.

	*brh*	*srh*	*β_2_*	*A2A*	*CXCR4*	*D3*	*H1*	*M2*	*S1P1*
*brh*	0.63 (100)								
*srh*	1.64 (30.0)	0.29 (100)							
*β_2_*	1.94 (21.5)	1.92 (25.5)	0.33 (100)						
*A2A*	2.41 (23.5)	2.34 (23.5)	2.00 (35.5)	0.60 (100)					
*CXCR4*	2.39 (22.0)	2.39 (21.5)	2.51 (24.0)	2.70 (22.0)	0.71 (100)				
*D3*	1.79 (28.0)	1.71 (20.5)	1.46 (38.0)	1.60 (32.5)	2.21 (27.5)	0.57 (100)			
*H1*	2.00 (20.0)	1.99 (23.0)	1.47 (36.5)	2.02 (34.0)	2.29 (25.0)	1.46 (35.5)			
*M2*	2.37 (22.5)	2.46 (20.5)	1.55 (29.5)	2.28 (27.0)	2.65 (23.0)	1.95 (33.0)	1.81 (37.5)		
*S1P1*	2.55 (21.5)	2.54 (23.5)	2.06 (27.0)	1.99 (29.5)	2.78 (22.0)	1.94 (26.5)	2.16 (24.5)	2.34 (27.0)	0.40 (100)

Amino acid sequence identity (%) is also shown in parenthesis. The range of each helix contained in a 200 residue 7TM bundle with Ballesteros & Weinstein numbering is as follows: helix I, 1.35–1.59 (25 a.a.); helix II, 2.38–2.67 (30 a.a.); helix III, 3.22–3.55 (34 a.a.); helix IV, 4.39–4.63 (25 a.a.); helix V, 5.36–5.65 (30 a.a.); helix VI, 6.29–6.60 (32 a.a.); helix VII, 7.32–7.55 (24 a.a.). brh and srh stand for bovine rhodopsin and squid rhodopsin, respectively.

**Figure 1:**
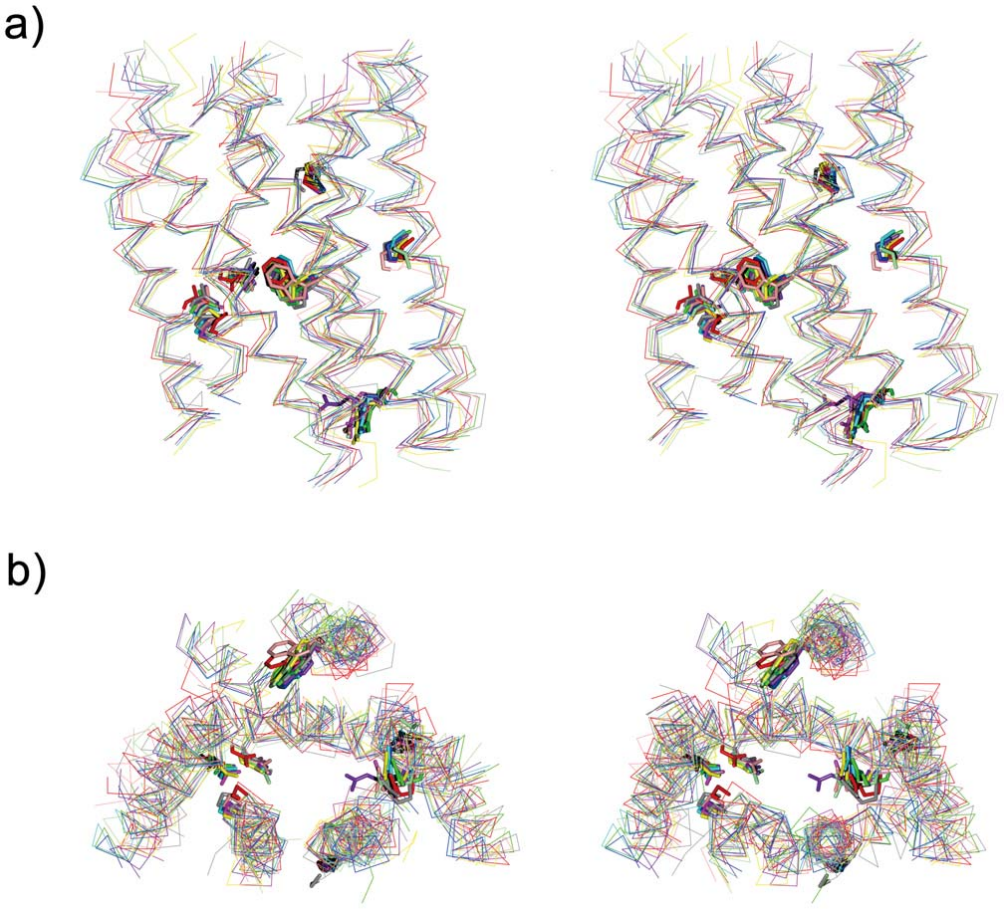
Stereo view of molecular overlay of 10 representative 7TM bundles of inactivated GPCRs of known structure. a) The extracellular side is facing upward while helix I is shown on the left. b) The 7TM bundles are viewed from the bottom of a). Seven highly conserved residues (*.50 s) are shown on the Cα backbone traces. The colors of receptors are red, bovine rhodopsin (1U19-A); pink, squid rhodopsin (2Z73-A); blue, β2 receptor (2RH1); cyan, β1 receptor (2VT4-B); yellow, A2A receptor (3EML); gray, CXCR4 receptor (3ODU-A); magenta, D3 receptor (3PBL-A); purple, H1 receptor (3RZE); green, M2 receptor (3UON); light green, S1P1 receptor (3V2Y).

All of the 200 residue 7TM bundles were superposed on that of the highest resolution inactivated form of β2-adrenergic receptor (PDB entry 2RH1) by a secondary structure matching (SSM) algorithm [12] implemented in Coot [13], from which 10 representative chains are shown in Figure 1. Different choices of the reference bundle to which all the others were fit did not affect the results of the current analysis.

**Figure 2:**
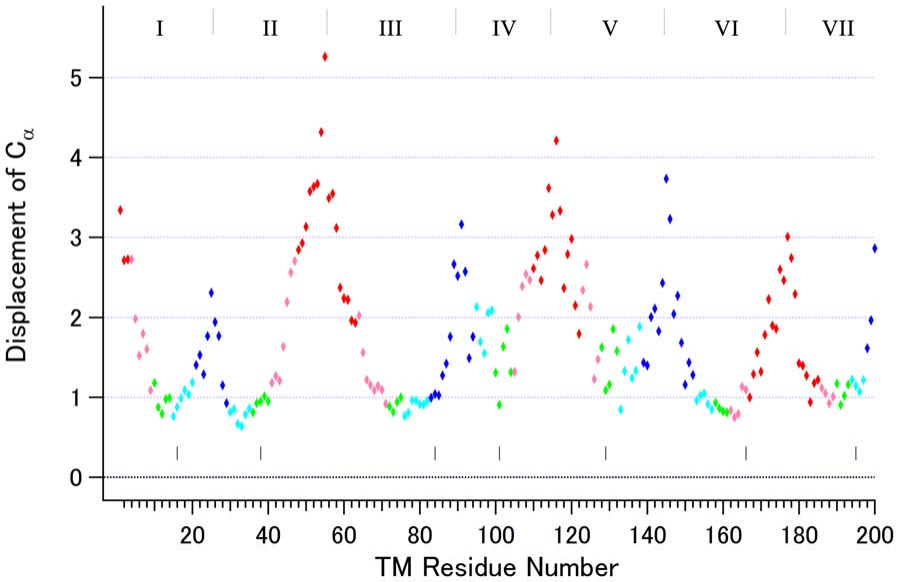
Averaged displacement (Å) of Cα atoms of 200 residues. Thirty-six pairwise deviations at each position were averaged and plotted against a serial number of residues in a 7TM bundle. The residues located in the same section (slab) are colored in red, section 1; pink, section 2; green, section 3; cyan, section 4; blue, section 5. The borders between the adjacent helices are shown with gray bars at the top of the panel, while the positions of seven *.50 residues are shown with short black bars near the zero line of the graph.

Using 9 representative chains (1U19-A for bovine rhodopsin, 2Z73-A for squid rhodopsin, 2RH1 for β2 receptor, 3EML for A2A receptor, 3ODU-A for CXCR4 receptor, 3PBL-A for D3 receptor, 3RZE for H1 receptor, 3UON for M2 receptor and 3V2Y for S1P1 receptor), deviations at 200 positions were calculated for all possible 36 pairs by CCP4 [14], demonstrating that the SSM fitting worked well because the Cα atoms of highly conserved residues (such as *.50s of Ballesteros & Weinstein numbering [15] for GPCRs, in which * is to be replaced with the number of helix) exhibited very small shifts among the chains from different receptors (Figure 2). For instance, an arginine residue (3.50) in the highly conserved D/ERY motif at the cytoplasmic end of helix III (Figure S1b, Supporting Information) exhibits an averaged deviation of 1.05 Å for the Cα position. On the other hand, the Cα of a glutamate residue (6.30) that was supposed to form the so-called “ionic lock” with the arginine residue (3.50) significantly differs (3.24 Å) between receptors. In fact, such an “ionic lock” is present only in 3 (bovine and squid rhodopsins, D3 receptor) out of 10 representative chains (2 chains do not have Glu/Asp at 6.30). These results indicate that SSM fitting clearly differentiates the conserved region from the variable region of the receptors. This finding is particularly important because it implies that fitting of the chains in the activated group to an inactivated reference is also appropriate to enable reasonable comparisons, as described later.

Overall similarity of the backbone traces from 10 receptors was then examined by rmsd calculation after superposition with the SSM algorithm. Using 50 chains (11 of bovine rhodopsin, 5 of squid rhodopsin, 5 of β2 receptor, 12 of β1 receptor, 6 of A2A receptor, 5 of CXCR4 receptor, 2 of D3 receptor, 1 of H1 receptor, 1 of M2 receptor and 2 of S1P1 receptor) of the inactivated form, 1225 pairwise values could be obtained. However, 7 chains of β1 receptor were excluded because they deviated substantially from other chains at the N-terminal part of helix I [16] or helix VI [17].

Then, from the remaining 43 chains, all 903 pairwise rmsd values were grouped into 53 receptor pairs and averaged for each of the pairs, of which the results for 43 pairs are shown in Table 1. For instance, an averaged rmsd among 11 chains of bovine rhodopsin was calculated to be 0.63 from 55 values. This value and others could have been even lower had we considered stricter criteria, such as excluding some chains of lower resolution structure. The standard deviations of the averaged values are less than 0.1 Å, except for brh-brh, srh-srh, β1-β1, A2A-A2A, CXCR4-CXCR4 pairs, indicating that the rmsds obtained between different receptors did not vary so much. It should be noted that the rmsd values shown here include the effect of mismatches between the amino acid sequence and structural alignments. Comparison of each helix indicated that such mismatches arose at helix II with CXCR4 receptor [7] and squid rhodopsin. Current analysis of the 7TM bundles also suggests that there are some other regions, including the extracellular side of helix IV [7], for which it is still difficult to determine if a large deviation for a particular receptor results either from the deformation of the helical structure or from the insertion/deletion of a single residue.

To reveal structural features of a receptor in terms of ligand binding specificity, sectional comparisons were performed for 10 receptors. Stability and diversity of 7TM structure vary among the sections stacked along the normal to the membrane plane [7], [18]. According to previous electron microscopic observation on two-dimensional crystals of bovine rhodopsin [19], it is reasonable to assume that the axis of helix IV is almost perpendicular to the membrane plane. In addition, secondary structure analysis by DSSP [20] indicated that helix IV of both adrenergic receptors and M2 receptor contains canonical α-helical turns throughout the 25 residues in the 7TM bundle.

Therefore, we divided a 7TM bundle into 5 sections, corresponding to 5 residues of helix IV per section (Figure S1, Supporting Information). Each section had similar thickness (about 7.5 Å) along the normal to the membrane plane, except sections 1 and 5 corresponding to the extracellular and cytoplasmic surface layers, respectively. Because of the different lengths of some helices, as mentioned above, neither of these 2 sections had a flat surface. The range of amino acid sequences of the 7 helices chosen in each section is summarized in Table S2 of the Supporting Information. The boundaries between sections 2/3, 3/4, and 4/5 are similar to those described previously [18].

Because section 1 is the most relevant region as the binding path for diffusible ligands from the extracellular space, one might expect that deviation among the receptors should be larger than for the other sections. This was found to be the case for most of the pairwise combinations, except for the pairs of adrenergic receptors and muscarinic receptors. The low rmsds between muscarinic and adrenergic receptors in this section are remarkable, especially compared with that of the other sections (see Table S3, Supporting Information).

**Figure 3:**
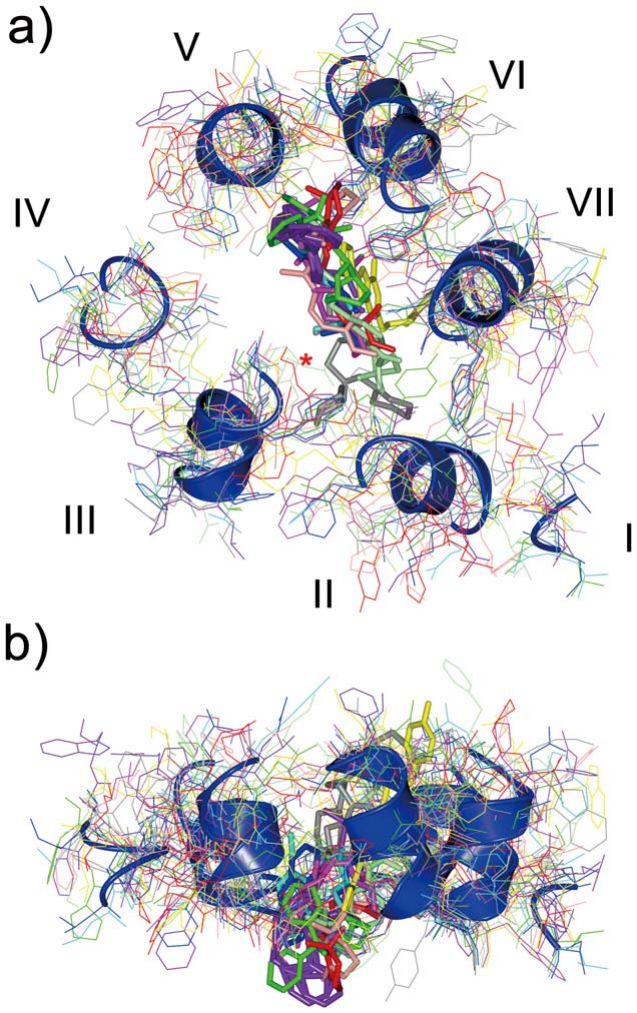
Molecular overlay of section 1 of 10 representative chains of inactivated GPCRs of known structure. All the polypeptides (thin lines) and bound ligands (thick lines) are viewed (a) perpendicularly (from the cytoplasmic side) or (b) horizontally (from the top of a), parallel to the membrane). Backbone ribbons of β2 receptor are also shown to clarify the position of the 7 helices. Colors of the side chains and ligands of each receptor are the same as in Figure 1. The Cα backbone trace of bovine rhodopsin is marked with a red asterisk in a).

In spite of the larger backbone deviation in section 1, the position of the bound ligand in each receptor is restricted within an overlapping space between helices II and V (Figure 3a). On the other hand, the ligand position varies significantly along the normal to the membrane plane. The ligands of rhodopsin, H1 receptor and M2 receptor reach down to the space corresponding to section 2, while the ligand of A2A receptor extends up to the extracellular surface of the 7TM bundle (Figure 3b).

**Figure 4:**
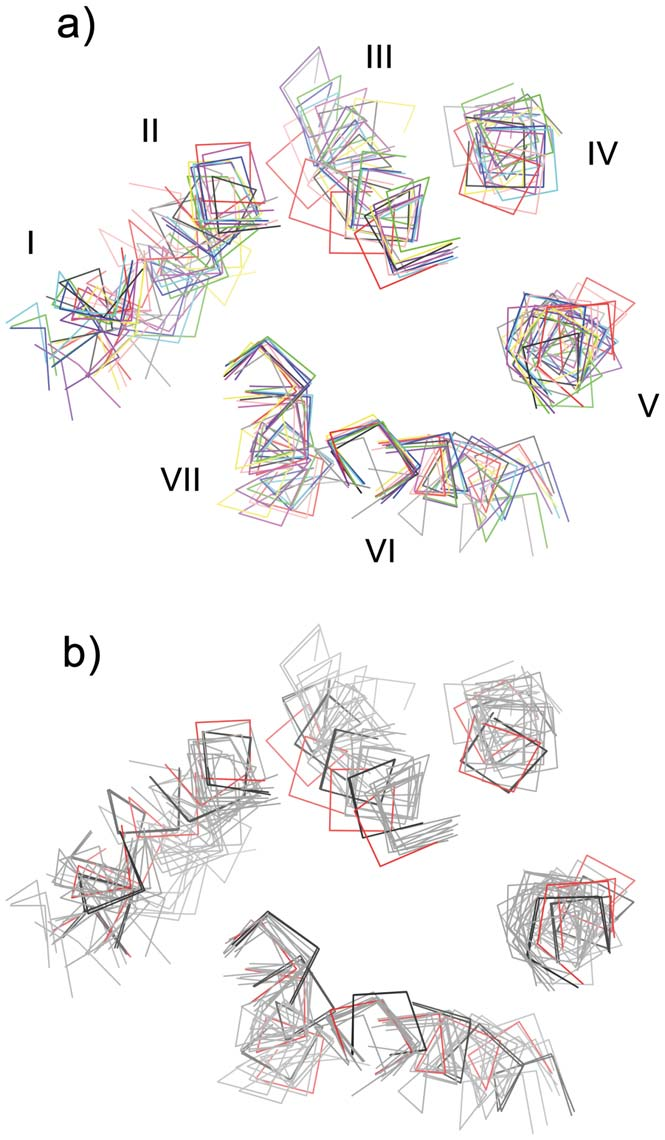
Projection view (from the cytoplasmic side) of molecular overlay of sections 1 and 2. a) 10 representative chains of inactivated GPCRs. b) the same inactive chains plus 2 chains of activated bovine rhodopsin. The colors in a) are the same as in Figure 1. In b), 1 inactivated chain (1U19-A) and 2 activated chains of bovine rhodopsin (PDB ID: 3PQR and 3PXO) are shown in red and black, respectively, while other chains are shown in gray.

The pocket for the ligands shown in Figure 3 is mostly composed of the side chains of section 1. However, we noticed that a short backbone trace of helix III of bovine rhodopsin was also visible. To clarify the unique arrangement of bovine rhodopsin in this region, a molecular overlay of the Cα trace of sections 1 and 2 of the representative chains of 10 receptors is shown in Figure 4.

The most remarkable finding is that only the Cα backbone of bovine rhodopsin deviates significantly from the middle to the extracellular side of helix III, roughly from 3.27 to 3.36. This feature was prominent even after overlaying all 50 inactivated chains. The most closely related GPCR of known structure, squid rhodopsin was slightly closer to bovine rhodopsin but only from 3.27 to 3.31. In addition, no chains deviated by as many as 3 helical turns toward the inside of the 7TM bundle at any of the other helices.

Position 3.36 is close to the boundary between sections 2 and 3, where deviations among the receptors were quite small (Figure 2). A unique feature of vertebrate rhodopsin in the amino acid sequence of helix III is the presence of Glu113 (3.28), the counterion to the protonated 11-*cis*-retinal Schiff base, the intrinsic inverse-agonist covalently bound to the side chain of Lys296 (7.43). The salt-bridge between Schiff base proton and deprotonated side chain of Glu113 is one of the constraints that force the protein moiety to be quiescent [21]. We suppose that acquisition of an acidic residue at 3.28 during molecular evolution might be responsible for the structural rearrangement of TM3 in vertebrate rhodopsin because invertebrate squid rhodopsin does not have glutamate or aspartate at this position.

Secondary structure analysis indicated that helix III adopts a regular α-helical structure with no obvious kink in each of the 10 receptors. Both bovine and squid rhodopsins have a Gly-Gly sequence in the middle of this helix although the position is shifted roughly 1 turn toward the extracellular side in squid rhodopsin. The tandem glycine, however, is not the cause of inward shift of helix III in rhodopsin because when this helix was analyzed in isolation, its shape was nearly invariant across receptors (Figure S2, Supporting Information). Thus the deviation in rhodopsin represents a rigid-body rearrangement within the 7TM bundle.

The inward displacement in the middle of helix III of inactivated bovine rhodopsin appears to have another implication in terms of photoactivation. Previous low temperature crystallographic studies trapping the early photoreaction intermediates demonstrated that the absorbed photon energy is initially stored in bathorhodopsin which is observable transiently in pico- to nanoseconds at room temperature and has a highly distorted all-*trans*-retinal chromophore [22]. Then during the thermal relaxation process of bathorhodopsin in nano- to microseconds, the energy is transmitted to the protein moiety as an outward deformation in the middle of helix III [23].

Considering the current analysis of backbone structure, it would be possible to conclude that the inward displacement of helix III in the mid to extracellular sections makes rhodopsin more constrained than the other GPCRs. In fact, the 7TM bundles of bovine rhodopsin with all-*trans*-retinal agonist bound (a mimic of the physiologically active intermediate formed in milliseconds) exhibit an arrangement of helix III similar to that observed in other GPCRs (Figure 4b). It should be also noted that, in addition to lateral displacement discussed here, a small backbone shift along the axis of helix III could contribute to the activation of rhodopsin, as described for A2A receptor [8] and discussed below.

## Discussion

To initiate the process of rhodopsin activation, photon energy is required to push the middle of helix III transiently out to a position similar to that observed in the other receptors. Conversely, such a primary process is not necessary for other receptors whose basal activity is not quenched to the exceptionally low level achieved in bovine rhodopsin. It is also possible that the distinct feature of the ligand binding sections in bovine rhodopsin correlates with the stability of the inactive conformation at the cytoplasmic surface. In this respect, it is particularly important to consider the presence of the D/ERY motif near the cytoplasmic end of helix III.

As noted above, the Cα position of the arginine (3.50) in this motif deviates only an average of 1 Å among all the receptor pairs of known inactive structures. Furthermore, Figure 2 indicates that the deviations in this motif are exceptionally small among the residues located in section 5 (colored blue). These findings are consistent with the idea that the position of this motif is critical for the activation of rhodopsin-like GPCRs.

The present study provides valuable information to enable a general scheme of activation to be drawn, because all the chains under consideration have identical helix lengths and are superposed on a single common reference chain. Although it has become evident that a major structural change in the 7TM bundle induced upon activation is the rigid-body movement of helix VI [24], [25] resulting in the binding of trimeric G protein [26], considerable variation was observed in the magnitude of the positional change of the cytoplasmic terminal residues of this helix (Figure S3, Supporting Information) depending on both the type of receptor and the presence of stabilizing partner such as nanobody [27] and trimeric Gαβγ [26]. The smaller changes in the cytoplasmic regions of helix V and VII, previously proposed to accompany activation [8], do not appear to be consistent among the receptors (bovine rhodopsin, β2 receptor and A2A receptor) for which both inactivated and activated chains are available.

As mentioned above, a small axial translation of helix III appears to occur toward the extracellular side upon activation of bovine rhodopsin and A2A receptor, and this is also consistent with the change observed for the β2 receptor based on the present superposition. Therefore, it appears possible that the axial shift of helix III and the large rigid body movement of helix VI are structurally linked in these receptors. It is noteworthy that mutations affecting the interaction between helices III and VI are known to cause constitutive activation of rhodopsin and other GPCRs. Such mutations have been found not only at the cytoplasmic surface sites, which possibly affects the ionic-lock between Arg(3.50) and Glu(6.30), but also at the middle of these helices [28], [29]. The latter findings are consistent with the recent proposal that the activation of β2 receptor accompanies a relative position change between the residues of helix III (3.40) and VI (6.44) interacting with each other [27]. The present analysis indicates that a similar change also occurs in bovine rhodopsin and A2A receptor between the residue at 3.36 and a conserved tryptophan (6.48). Exceptionally low activity of bovine rhodopsin would result, at least partly, from the unique rigid-body arrangement of helix III as demonstrated above, which would restrict the movements of these residues and the bound inverse-agonist 11-*cis*-retinal. In summary, stabilization of the interface between helices III and VI at the central part of the transmembrane region is likely to suppress the movement of these helices, and thereby, restrain the cytoplasmic surface of receptor to the inactive conformation.

The present method can easily be applied to survey the growing number of experimental structures of rhodopsin-like GPCRs. In addition, it will aid other computational studies, such as simulation of the activation process and homology based modeling of the receptors with unknown structure, by providing some statistical constraints on the positional deviation allowed for each of the residues in the 7TM bundle during the calculations. For example, further accumulation of experimental structures of a variety of activated receptors would highlight certain regions within the bundle that exhibit little positional differences before and after activation. Subsequently, the identified regions would be treated as a common scaffold during simulation of the activation process. Similarly, the data of the positional deviations among the receptors, when combined with the data of sequence identities and/or similarities, is beneficial for modeling the 7TM bundles of receptors that have not yet been crystallized. For these applications, it is also important to identify and consider the possible contribution of internal hydrogen bonds including the water molecules conserved among the receptors [30], [31].

### Note Added in Proof

After revision of this paper, four new entries (five chains) in the PDB have been released, namely, two inactivated chains of human kappa opioid receptor (PDBID: 4DJH), one inactivated chain of mouse mu opioid receptor (4DKL), and two inactivated chains of A2A receptor (3UZA, 3UZC). These structures confirm the findings described in this paper. Updates to Table 1 and Tables S1, S2, and S3 are available at http://www.gses.jp/7tmsp/.

## Materials and Methods

Crystallographic entries for GPCRs were obtained from Protein Data Bank. They were classified into two categories: an inactivated group (antagonist bound, inverse-agonist bound) and an activated group (agonist bound, ligand free). After separating the polypeptide chains from the entries, SSM superposition was performed with Coot [13] for the representative inactivated chains of several receptors. Then, the range of each helix consisting of a 7TM bundle was determined as shown in the footnote of Table 1, guided by the results of multiple sequence alignment performed at GPCRDB. A 7TM bundle of 200 residues was successfully extracted from 80 chains and superposed on that of the highest resolution inactivated form of β2-adrenergic receptor (PDB entry 2RH1).

Rmsds were calculated with Discovery Studio Visualizer (Accelrys Inc.). The values were identical to that obtained with Chimera [32]. Deviations at 200 residue positions were calculated by CCP4 [10] for all possible 36 pairs of 9 receptors. β1 receptor was not considered because its backbone structure is quite similar to that of β2 receptor, as shown in Table S3 of Supporting Information. Secondary structure of each receptor was analyzed by DSSP [20]. Figures 1, 3, 4, S1, S2 and S3 including the parallel (wall-eye) stereo pictures were prepared using CCP4MG [33].

## Supporting Information

Figure S1 **Sectional comparison of 7TM bundles.** a) Stereo view of molecular overlay of 5 sections from 10 representative chains of inactivated GPCRs. Cα traces are viewed from the same direction as Figure 1a. b) Snake model of 7TM bundle showing the range of residues in 5 sections. Two to three Ballesteros & Weinstein numbers are shown for each helix. The position of *.50 residue in each helix and of the D/ERY motif in helix III are indicated with a thick circle and a white rectangle, respectively. The colors of five sections are the same as in Figure 2.
(TIF)

Figure S2 **Comparison of helix III.** a) Stereo view of molecular overlay of helix III within 7TM bundles of the representative chains of 10 receptors: bovine rhodopsin (red), squid rhodopsin (pink), β2 receptor (blue), β1 receptor (cyan), A2A receptor (yellow), CXCR4 receptor (grey), D3 receptor (magenta), H1 receptor (purple), M2 receptor (green) and S1P1 receptor (light green). b) Stereo view of molecular overlay of isolated helix III from the representative chains of 10 receptors after superposition to isolated helix III of β2 receptor.
(TIF)

Figure S3 **Comparison of structural change of helix VI.** Stereo view of molecular overlay of helix VI within 7TM bundles: inactivated (red, PDB ID: 1U19-A) and activated (pink, PDB ID: 3PXO) bovine rhodopsin, inactivated (blue, PDB ID: 2RH1) and activated (cyan, PDB ID: 3SN6) β2 receptor, inactivated (yellow, PDB ID: 3EML) and activated (lemon, PDB ID: 3QAK) A2A receptor.
(TIF)

Table S1 **Full list of the chains considered.** The chains used in this work are shown as 200 in the 7TM column. Abbreviations Ab: antibody bound, Nb: nanobody bound, T4L: T4 lysozyme merged.
(DOC)

Table S2 **The range of amino acid sequences of 7 helices chosen in 5 sections.** Each of H, s, e, BW, brh and srh stands for helix number, start residue number, end residue number, Ballesteros & Weinstein number, bovine rhodopsin and squid rhodopsin, respectively.
(DOC)

Table S3 **Sequence identity, similarity and rmsd among the representative chains of 10 receptors, for all 200 residues and for each of 5 sections.**
(DOC)
