# Supplementary figures and images for: Comparative Analysis of the Heptahelical Transmembrane Bundles of G Protein-Coupled Receptors
Source: PLoS One. 2012 Apr 24;7(4):e35802. doi: 10.1371/journal.pone.0035802 (PMC3335790; doi:10.1371/journal.pone.0035802)

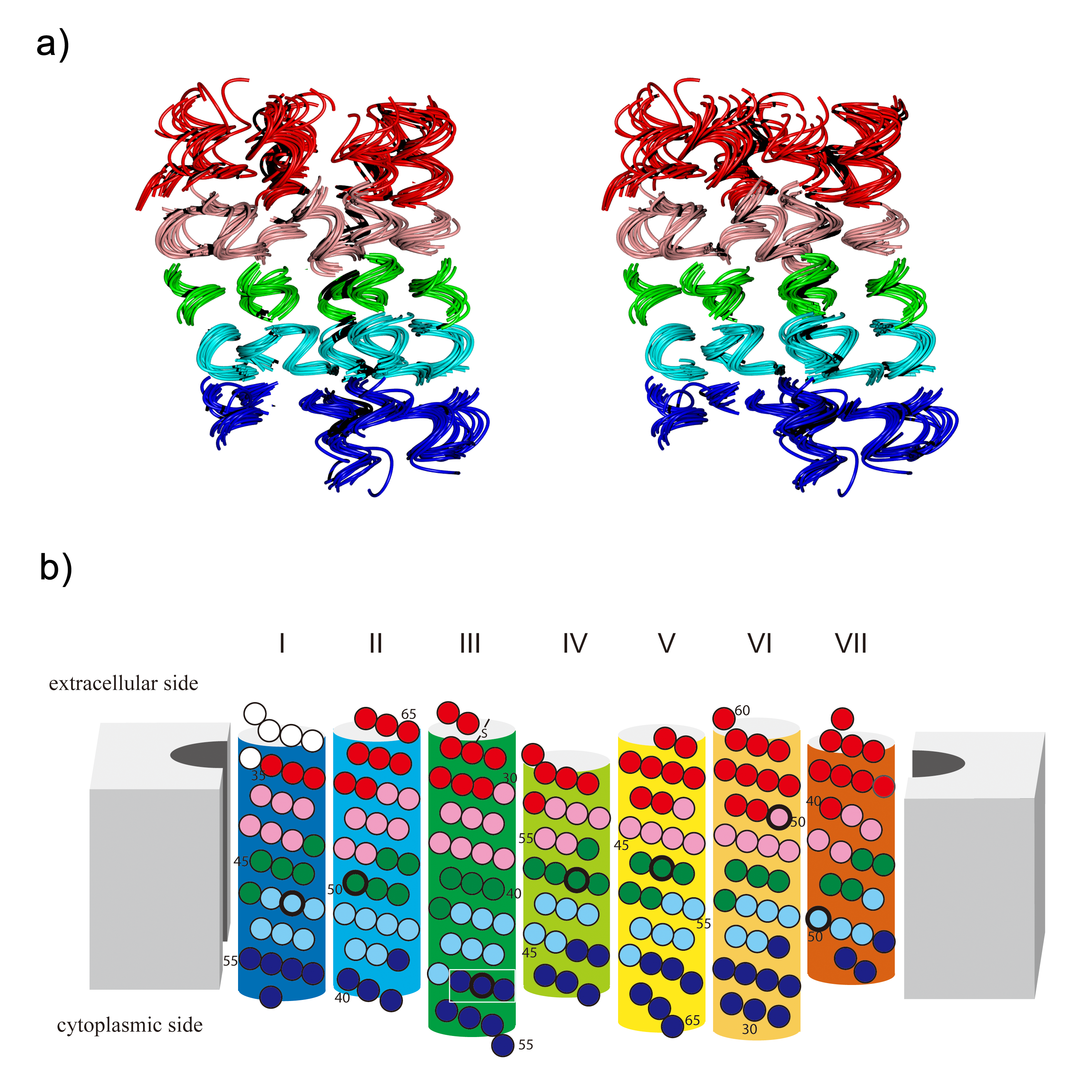

Supplement: Figure S1 — Sectional comparison of 7TM bundles. a) Stereo view of molecular overlay of 5 sections from 10 representative chains of inactivated GPCRs. Cα traces are viewed from the same direction as Figure 1a. b) Snake model of 7TM bundle showing the range of residues in 5 sections. Two to three Ballesteros & Weinstein numbers are shown for each helix. The position of *.50 residue in each helix and of the D/ERY motif in helix III are indicated with a thick circle and a white rectangle, respectively. The colors of five sections are the same as in Figure 2. (TIF) [file pone.0035802.s001.tif]

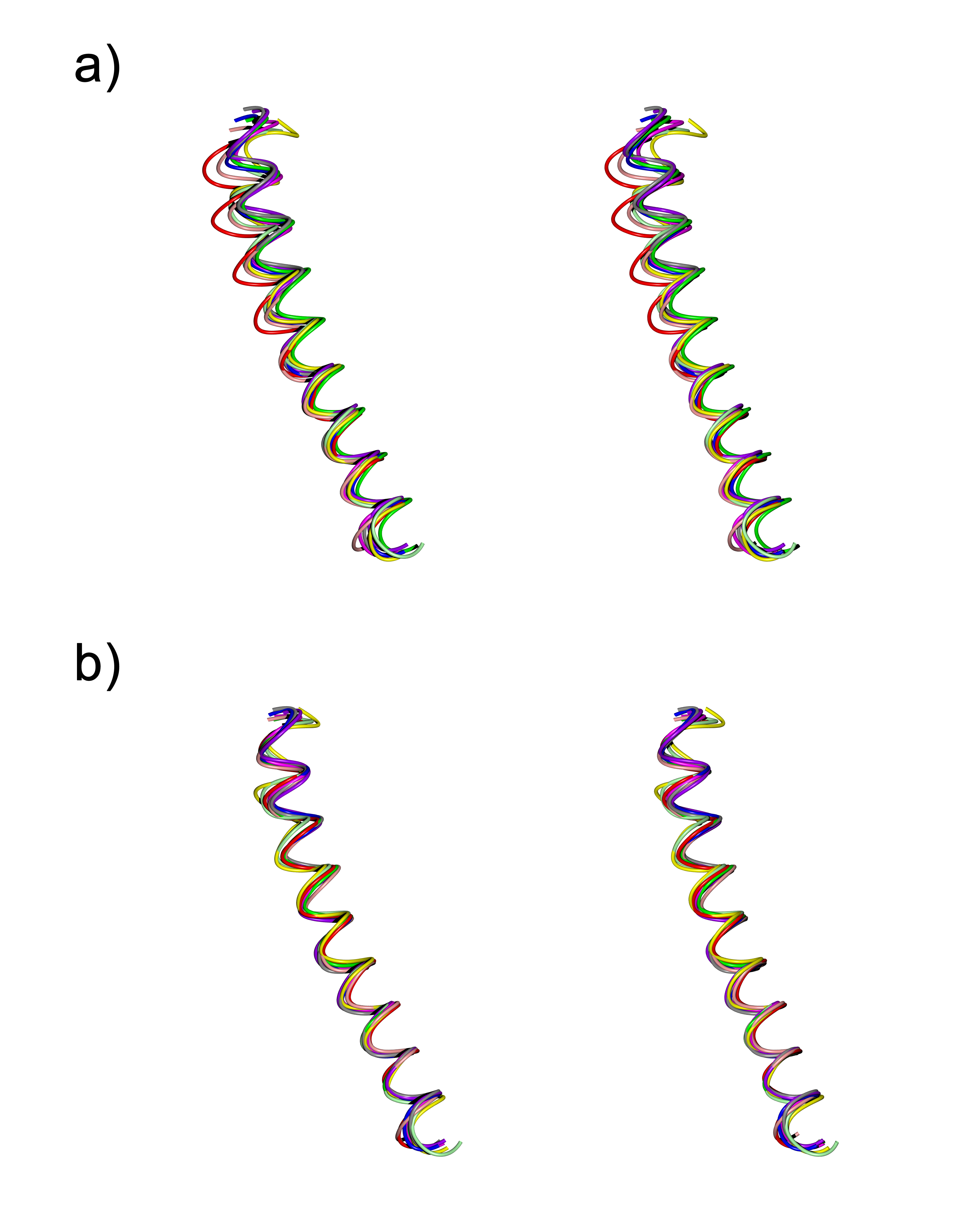

Supplement: Figure S2 — Comparison of helix III. a) Stereo view of molecular overlay of helix III within 7TM bundles of the representative chains of 10 receptors: bovine rhodopsin (red), squid rhodopsin (pink), β2 receptor (blue), β1 receptor (cyan), A2A receptor (yellow), CXCR4 receptor (grey), D3 receptor (magenta), H1 receptor (purple), M2 receptor (green) and S1P1 receptor (light green). b) Stereo view of molecular overlay of isolated helix III from the representative chains of 10 receptors after superposition to isolated helix III of β2 receptor. (TIF) [file pone.0035802.s002.tif]

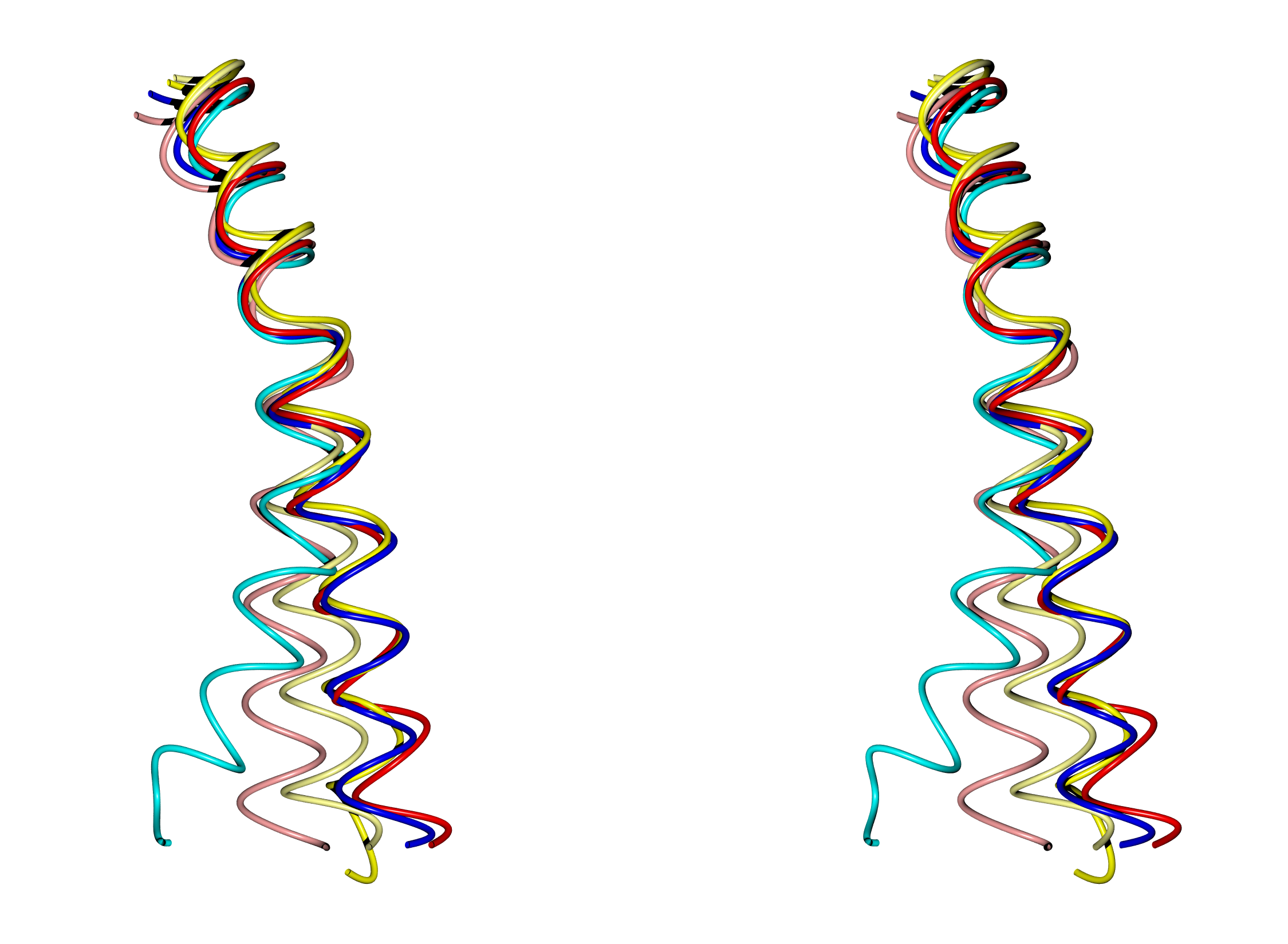

Supplement: Figure S3 — Comparison of structural change of helix VI. Stereo view of molecular overlay of helix VI within 7TM bundles: inactivated (red, PDB ID: 1U19-A) and activated (pink, PDB ID: 3PXO) bovine rhodopsin, inactivated (blue, PDB ID: 2RH1) and activated (cyan, PDB ID: 3SN6) β2 receptor, inactivated (yellow, PDB ID: 3EML) and activated (lemon, PDB ID: 3QAK) A2A receptor. (TIF) [file pone.0035802.s003.tif]
