# Supplementary material for: Comparative Analysis of the Heptahelical Transmembrane Bundles of G Protein-Coupled Receptors
Source: PLoS One. 2012 Apr 24;7(4):e35802. doi: 10.1371/journal.pone.0035802 (PMC3335790; doi:10.1371/journal.pone.0035802)
Supplement: Table S1 — Full list of the chains considered. The chains used in this work are shown as 200 in the 7TM column. Abbreviations Ab: antibody bound, Nb: nanobody bound, T4L: T4 lysozyme merged. (DOC) [file pone.0035802.s004.doc]

Table S1: Full list of the chains considered. The chains used in this work are shown as 200 in the 7TM column.

Abbreviations

Ab: antibody bound, Nb: nanobody bound, T4L: T4 lysozyme merged

| **receptor** | **PDB** | **7TM** | **ligand** | **remarks** |
| --- | --- | --- | --- | --- |
| **inactivated** |  |  |  |  |
| rhodopsin | 1GZM | 200 (A) | 11-cis-retinal | bovine |
|  |  | 200 (B) | 11-cis-retinal | bovine |
|  | 1U19 | 200 (A) | 11-cis-retinal | bovine |
|  |  | 200 (B) | 11-cis-retinal | bovine |
|  | 2I35 | 200 (A) | 11-cis-retinal | bovine |
|  | 2I36 | 200 (A) | 11-cis-retinal | bovine |
|  |  | 196 (B) | 11-cis-retinal | bovine |
|  |  | 200 (C) | 11-cis-retinal | bovine |
|  | 2J4Y | 200 (A) | 11-cis-retinal | bovine |
|  |  | 200 (B) | 11-cis-retinal | bovine |
|  | 2PED | 200 (A) | 9-cis-retinal | bovine |
|  |  | 200 (B) | 9-cis-retinal | bovine |
|  | 2Z73 | 200 (A) | 11-cis-retinal | squid |
|  |  | 200 (B) | 11-cis-retinal | squid |
|  | 2ZIY | 200 (A) | 11-cis-retinal | squid |
|  | 3AYN | 200 (A) | 9-cis-retinal | squid |
|  |  | 200 (B) | 9-cis-retinal | squid |
| beta2 adrenergic | 2RH1 | 200 (A) | carazorol | human, T4L |
|  | 2R4R | 161 (A) | invisible | human, Ab |
|  | 2R4S | 161 (A) | invisible | human, Ab |
|  | 3D4S | 200 (A) | timolol | human, 1 mutated(3.41), T4L |
|  | 3KJ6 | 167 (A) | invisible | human, Ab, methylated |
|  | 3NY8 | 200 (A) | ICI 118551 | human, 1 mutated(3.41), T4L |
|  | 3NY9 | 200 (A) | JSZ | human, 1 mutated(3.41), T4L |
|  | 3NYA | 200 (A) | alprenolol | human, 1 mutated(3.41), T4L |
| beta1 adrenergic | 2VT4 | 200 (A) | cyanopindolol | turkey, TM1 N-term bent |
|  |  | 200 (B) | cyanopindolol | turkey |
|  |  | 200 (C) | cyanopindolol | turkey |
|  |  | 200 (D) | cyanopindolol | turkey, TM1 N-term bent |
|  | 2YCW | 200 (A) | carazorol | turkey, TM6 N-term bent |
|  |  | 200 (B) | carazorol | turkey |
|  | 2YCY | 200 (A) | cyanopindolol | turkey, TM1 N-term bent |
|  |  | 200 (B) | cyanopindolol | turkey |
|  | 2YCX | 200 (A) | cyanopindolol | turkey, TM6 N-term bent |
|  |  | 200 (B) | cyanopindolol | turkey, TM6 N-term bent |
|  | 2YCZ | 200 (A) | iodocyanopindolol | turkey |
|  |  | 200 (B) | iodocyanopindolol | turkey, TM6 N-term bent |
| Adenosine A2A | 3EML | 200 (A) | ZM241385 | human, T4L |
|  | 3PWH | 200 (A) | ZM241385 | human, 8 mutated |
|  | 3REY | 200 (A) | XAC | human, 8 mutated |
|  | 3RFM | 200 (A) | caffeine | human, 8 mutated |
|  | 3VG9 | 200 (A) | ZM241385 | human, Ab |
|  | 3VGA | 200 (A) | ZM241385 | human, Ab |
| CXCR4 chemokine | 3ODU | 200 (A) | It1t | human, T4L |
|  |  | 200 (B) | It1t | human, T4L |
|  | 3OE8 | 200 (A) | It1t | human, T4L |
|  |  | 200 (B) | It1t | human, T4L |
|  |  | 200 (C) | It1t | human, T4L |
|  | 3OE9 | 196 (A) | It1t | human, T4L |
|  |  | 196 (B) | It1t | human, T4L |
|  | 3OE6 | 198 (A) | It1t | human, T4L |
|  | 3OE0 | 199 (A) | CVX15 peptide | human, T4L |
| Dopamine D3 | 3PBL | 200 (A) | eticlopride | human, T4L |
|  |  | 200 (B) | eticlopride | human, T4L |
| Histamine H1 | 3RZE | 200 (A) | doxepin | human, T4L |
| M2 Muscarinic | 3UON | 200 (A) | QNB | human, T4L |
| M3 Muscarinic | 4DAJ | 197 (A) | tiotropium | rat, T4L |
|  |  | 197 (A) | tiotropium | rat, T4L |
|  |  | 197 (A) | tiotropium | rat, T4L |
|  |  | 198 (A) | tiotropium | rat, T4L |
| S1P1 | 3V2W | 200 (A) | ML056 | human, T4L |
|  | 3V2Y | 200 (A) | ML056 | human, T4L |
|  |  |  |  |  |
| **activated** |  |  |  |  |
| rhodopsin | 2G87 | 200 (A) | all-trans-retinal | bovine |
|  |  | 200 (B) | all-trans-retinal | bovine |
|  | 2HPY | 200 (A) | all-trans-retinal | bovine |
|  |  | 200 (B) | all-trans-retinal | bovine |
|  | 2I37 | 199 (A) | invisible | bovine |
|  |  | 200 (B) | invisible | bovine |
|  |  | 195 (C) | invisible | bovine |
|  | 3CAP | 200 (A) | none | bovine |
|  |  | 200 (B) | none | bovine |
|  | 3DQB | 200 (A) | none | bovine, Gt-peptide |
|  | 3PQR | 200 (A) | all-trans-retinal | bovine, Gt-peptide |
|  | 3PXO | 200 (A) | all-trans-retinal | bovine |
|  | 2X72 | 200 (A) | all-trans-retinal | bovine, 1 mutated, Gt-peptide |
|  | 4A4M | 200 (A) | all-trans-retinal | bovine, 1 mutated, Gt-peptide |
|  | 3AYM | 200 (A) | all-trans-retinal | squid |
|  |  | 200 (B) | all-trans-retinal | squid |
| beta2 adrenergic | 3P0G | 200 (A) | BI-167107 | human, Nb, T4L |
|  | 3PDS | 200 (A) | FAUC50 | human, 1 mutated, T4L |
|  | 3SN6 | 200 (A) | BI-167107 | human, 1 mutated, Gs, Nb, T4L |
| beta1 adrenergic | 2Y00 | 200 (A) | dobutamin | turkey |
|  |  | 200 (B) | dobutamin | turkey |
|  | 2Y01 | 200 (A) | dobutamin | turkey |
|  |  | 200 (B) | dobutamin | turkey |
|  | 2Y02 | 200 (A) | carmoterol | turkey |
|  |  | 200 (B) | carmoterol | turkey |
|  | 2Y03 | 200 (A) | isoprenaline | turkey |
|  |  | 200 (B) | isoprenaline | turkey |
|  | 2Y04 | 200 (A) | salbutamol | turkey |
|  |  | 200 (B) | salbutamol | turkey |
| Adenosine A2A | 3QAK | 200 (A) | UK-432097 | human, T4L |
|  | 2YDO | 200 (A) | adenosine | human, 4 mutated |
|  | 2YDV | 200 (A) | NECA | human, 4 mutated |
