# Supplementary material for: Comparative Analysis of the Heptahelical Transmembrane Bundles of G Protein-Coupled Receptors
Source: PLoS One. 2012 Apr 24;7(4):e35802. doi: 10.1371/journal.pone.0035802 (PMC3335790; doi:10.1371/journal.pone.0035802)
Supplement: Table S2 — The range of amino acid sequences of 7 helices chosen in 5 sections. Each of H, s, e, BW, brh and srh stands for helix number, start residue number, end residue number, Ballesteros & Weinstein number, bovine rhodopsin and squid rhodopsin, respectively. (DOC) [file pone.0035802.s005.doc]

Table S2: The range of amino acid sequences of 7 helices chosen in 5 sections.

Each of H, s, e, BW, brh and srh stands for helix number, start residue number, end residue number, Ballesteros & Weinstein number, bovine rhodopsin and squid rhodopsin, respectively.

| Sect. |  | H | s | e | H | s | e | H | s | e | H | s | E | H | s | e | H | s | e | H | s | e |
| --- | --- | --- | --- | --- | --- | --- | --- | --- | --- | --- | --- | --- | --- | --- | --- | --- | --- | --- | --- | --- | --- | --- |
| 1 | BW | 1 | 35 | 37 | 2 | 60 | 67 | 3 | 22 | 29 | 4 | 59 | 63 | 5 | 36 | 43 | 6 | 51 | 60 | 7 | 32 | 40 |
| 1 | brh | 1 | 40 | 42 | 2 | 93 | 100 | 3 | 107 | 114 | 4 | 170 | 174 | 5 | 201 | 208 | 6 | 268 | 277 | 7 | 285 | 293 |
| 1 | srh | 1 | 37 | 39 | 2 | 90 | 97 | 3 | 105 | 112 | 4 | 169 | 173 | 5 | 198 | 205 | 6 | 277 | 286 | 7 | 294 | 302 |
| 1 | β2 | 1 | 36 | 38 | 2 | 89 | 96 | 3 | 103 | 110 | 4 | 167 | 171 | 5 | 197 | 204 | 6 | 289 | 298 | 7 | 305 | 313 |
| 1 | β1 | 1 | 44 | 46 | 2 | 97 | 104 | 3 | 111 | 118 | 4 | 175 | 179 | 5 | 205 | 212 | 6 | 306 | 315 | 7 | 322 | 330 |
| 1 | A2A | 1 | 9 | 11 | 2 | 62 | 69 | 3 | 74 | 81 | 4 | 138 | 142 | 5 | 175 | 182 | 6 | 249 | 258 | 7 | 267 | 275 |
| 1 | CXCR4 | 1 | 41 | 43 | 2 | 94 | 101 | 3 | 106 | 113 | 4 | 170 | 174 | 5 | 197 | 204 | 6 | 255 | 264 | 7 | 281 | 289 |
| 1 | D3 | 1 | 32 | 34 | 2 | 85 | 92 | 3 | 100 | 107 | 4 | 167 | 171 | 5 | 186 | 193 | 6 | 345 | 354 | 7 | 362 | 370 |
| 1 | H1 | 1 | 30 | 32 | 2 | 83 | 90 | 3 | 97 | 104 | 4 | 161 | 165 | 5 | 188 | 195 | 6 | 431 | 440 | 7 | 447 | 455 |
| 1 | M2 | 1 | 26 | 28 | 2 | 79 | 86 | 3 | 93 | 100 | 4 | 157 | 161 | 5 | 184 | 191 | 6 | 403 | 412 | 7 | 419 | 427 |
| 1 | S1P1 | 1 | 48 | 50 | 2 | 101 | 108 | 3 | 114 | 121 | 4 | 177 | 181 | 5 | 199 | 206 | 6 | 272 | 281 | 7 | 290 | 298 |
|  |  |  |  |  |  |  |  |  |  |  |  |  |  |  |  |  |  |  |  |  |  |  |
| 2 | BW | 1 | 38 | 43 | 2 | 53 | 59 | 3 | 30 | 37 | 4 | 54 | 58 | 5 | 44 | 48 | 6 | 46 | 50 | 7 | 41 | 44 |
| 2 | brh | 1 | 43 | 48 | 2 | 86 | 92 | 3 | 115 | 122 | 4 | 165 | 169 | 5 | 209 | 213 | 6 | 263 | 267 | 7 | 294 | 297 |
| 2 | srh | 1 | 40 | 45 | 2 | 83 | 89 | 3 | 113 | 120 | 4 | 164 | 168 | 5 | 206 | 210 | 6 | 272 | 276 | 7 | 303 | 306 |
| 2 | β2 | 1 | 39 | 44 | 2 | 82 | 88 | 3 | 111 | 118 | 4 | 162 | 166 | 5 | 205 | 209 | 6 | 284 | 288 | 7 | 314 | 317 |
| 2 | β1 | 1 | 47 | 52 | 2 | 90 | 96 | 3 | 119 | 126 | 4 | 170 | 174 | 5 | 213 | 217 | 6 | 301 | 305 | 7 | 331 | 334 |
| 2 | A2A | 1 | 12 | 17 | 2 | 55 | 61 | 3 | 82 | 89 | 4 | 133 | 137 | 5 | 183 | 187 | 6 | 244 | 248 | 7 | 276 | 279 |
| 2 | CXCR4 | 1 | 44 | 49 | 2 | 87 | 93 | 3 | 114 | 121 | 4 | 165 | 169 | 5 | 205 | 209 | 6 | 250 | 254 | 7 | 290 | 293 |
| 2 | D3 | 1 | 35 | 40 | 2 | 78 | 84 | 3 | 108 | 115 | 4 | 162 | 166 | 5 | 194 | 198 | 6 | 340 | 344 | 7 | 371 | 374 |
| 2 | H1 | 1 | 33 | 38 | 2 | 76 | 82 | 3 | 105 | 112 | 4 | 156 | 160 | 5 | 196 | 200 | 6 | 426 | 430 | 7 | 456 | 459 |
| 2 | M2 | 1 | 29 | 34 | 2 | 72 | 78 | 3 | 101 | 108 | 4 | 152 | 156 | 5 | 192 | 196 | 6 | 398 | 402 | 7 | 428 | 431 |
| 2 | S1P1 | 1 | 51 | 56 | 2 | 94 | 100 | 3 | 122 | 129 | 4 | 172 | 176 | 5 | 207 | 211 | 6 | 267 | 271 | 7 | 299 | 302 |
|  |  |  |  |  |  |  |  |  |  |  |  |  |  |  |  |  |  |  |  |  |  |  |
| 3 | BW | 1 | 44 | 48 | 2 | 48 | 52 | 3 | 38 | 41 | 4 | 49 | 53 | 5 | 49 | 53 | 6 | 42 | 45 | 7 | 45 | 48 |
| 3 | brh | 1 | 49 | 53 | 2 | 81 | 85 | 3 | 123 | 126 | 4 | 160 | 164 | 5 | 214 | 218 | 6 | 259 | 262 | 7 | 298 | 301 |
| 3 | srh | 1 | 46 | 50 | 2 | 78 | 82 | 3 | 121 | 124 | 4 | 159 | 163 | 5 | 211 | 215 | 6 | 268 | 271 | 7 | 307 | 310 |
| 3 | β2 | 1 | 45 | 49 | 2 | 77 | 81 | 3 | 119 | 122 | 4 | 157 | 161 | 5 | 210 | 214 | 6 | 280 | 283 | 7 | 318 | 321 |
| 3 | β1 | 1 | 53 | 57 | 2 | 85 | 89 | 3 | 127 | 130 | 4 | 165 | 169 | 5 | 218 | 222 | 6 | 297 | 300 | 7 | 335 | 338 |
| 3 | A2A | 1 | 18 | 22 | 2 | 50 | 54 | 3 | 90 | 93 | 4 | 128 | 132 | 5 | 188 | 192 | 6 | 240 | 243 | 7 | 280 | 283 |
| 3 | CXCR4 | 1 | 50 | 54 | 2 | 82 | 86 | 3 | 122 | 125 | 4 | 160 | 164 | 5 | 210 | 214 | 6 | 246 | 249 | 7 | 294 | 297 |
| 3 | D3 | 1 | 41 | 45 | 2 | 73 | 77 | 3 | 116 | 119 | 4 | 157 | 161 | 5 | 199 | 203 | 6 | 336 | 339 | 7 | 375 | 378 |
| 3 | H1 | 1 | 39 | 43 | 2 | 71 | 75 | 3 | 113 | 116 | 4 | 151 | 155 | 5 | 201 | 205 | 6 | 422 | 425 | 7 | 460 | 463 |
| 3 | M2 | 1 | 35 | 39 | 2 | 67 | 71 | 3 | 109 | 112 | 4 | 147 | 151 | 5 | 197 | 201 | 6 | 394 | 397 | 7 | 432 | 435 |
| 3 | S1P1 | 1 | 57 | 61 | 2 | 89 | 93 | 3 | 130 | 133 | 4 | 167 | 171 | 5 | 212 | 216 | 6 | 263 | 266 | 7 | 303 | 306 |
|  |  |  |  |  |  |  |  |  |  |  |  |  |  |  |  |  |  |  |  |  |  |  |
|  | BW | 1 | 49 | 54 | 2 | 42 | 47 | 3 | 42 | 48 | 4 | 44 | 48 | 5 | 54 | 59 | 6 | 37 | 41 | 7 | 49 | 52 |
| 4 | brh | 1 | 54 | 59 | 2 | 75 | 80 | 3 | 127 | 133 | 4 | 155 | 159 | 5 | 219 | 224 | 6 | 254 | 258 | 7 | 302 | 305 |
| 4 | srh | 1 | 51 | 56 | 2 | 72 | 77 | 3 | 125 | 131 | 4 | 154 | 158 | 5 | 216 | 221 | 6 | 263 | 267 | 7 | 311 | 314 |
| 4 | β2 | 1 | 50 | 55 | 2 | 71 | 76 | 3 | 123 | 129 | 4 | 152 | 156 | 5 | 215 | 220 | 6 | 275 | 279 | 7 | 322 | 325 |
| 4 | β1 | 1 | 58 | 63 | 2 | 79 | 84 | 3 | 131 | 137 | 4 | 160 | 164 | 5 | 223 | 228 | 6 | 292 | 296 | 7 | 339 | 342 |
| 4 | A2A | 1 | 23 | 28 | 2 | 44 | 49 | 3 | 94 | 100 | 4 | 123 | 127 | 5 | 193 | 198 | 6 | 235 | 239 | 7 | 284 | 287 |
| 4 | CXCR4 | 1 | 55 | 60 | 2 | 76 | 81 | 3 | 126 | 132 | 4 | 155 | 159 | 5 | 215 | 220 | 6 | 241 | 245 | 7 | 298 | 301 |
| 4 | D3 | 1 | 46 | 51 | 2 | 67 | 72 | 3 | 120 | 126 | 4 | 152 | 156 | 5 | 204 | 209 | 6 | 331 | 335 | 7 | 379 | 382 |
| 4 | H1 | 1 | 44 | 49 | 2 | 65 | 70 | 3 | 117 | 123 | 4 | 146 | 150 | 5 | 206 | 211 | 6 | 417 | 421 | 7 | 464 | 467 |
| 4 | M2 | 1 | 40 | 45 | 2 | 61 | 66 | 3 | 113 | 119 | 4 | 142 | 146 | 5 | 202 | 207 | 6 | 389 | 393 | 7 | 436 | 439 |
| 4 | S1P1 | 1 | 62 | 67 | 2 | 83 | 88 | 3 | 134 | 140 | 4 | 162 | 166 | 5 | 217 | 222 | 6 | 258 | 262 | 7 | 307 | 310 |
|  |  |  |  |  |  |  |  |  |  |  |  |  |  |  |  |  |  |  |  |  |  |  |
|  | BW | 1 | 55 | 59 | 2 | 38 | 41 | 3 | 49 | 55 | 4 | 39 | 43 | 5 | 60 | 65 | 6 | 29 | 36 | 7 | 53 | 55 |
| 5 | brh | 1 | 60 | 64 | 2 | 71 | 74 | 3 | 134 | 140 | 4 | 150 | 154 | 5 | 225 | 230 | 6 | 246 | 253 | 7 | 306 | 308 |
| 5 | srh | 1 | 57 | 61 | 2 | 68 | 71 | 3 | 132 | 138 | 4 | 149 | 153 | 5 | 222 | 227 | 6 | 255 | 262 | 7 | 315 | 317 |
| 5 | β2 | 1 | 56 | 60 | 2 | 67 | 70 | 3 | 130 | 136 | 4 | 147 | 151 | 5 | 221 | 226 | 6 | 267 | 274 | 7 | 326 | 328 |
| 5 | β1 | 1 | 64 | 68 | 2 | 75 | 78 | 3 | 138 | 144 | 4 | 155 | 159 | 5 | 229 | 234 | 6 | 284 | 291 | 7 | 343 | 345 |
| 5 | A2A | 1 | 29 | 33 | 2 | 40 | 43 | 3 | 101 | 107 | 4 | 118 | 122 | 5 | 199 | 204 | 6 | 227 | 234 | 7 | 288 | 290 |
| 5 | CXCR4 | 1 | 61 | 65 | 2 | 72 | 75 | 3 | 133 | 139 | 4 | 150 | 154 | 5 | 221 | 226 | 6 | 233 | 240 | 7 | 302 | 304 |
| 5 | D3 | 1 | 52 | 56 | 2 | 63 | 66 | 3 | 127 | 133 | 4 | 147 | 151 | 5 | 210 | 215 | 6 | 323 | 330 | 7 | 383 | 385 |
| 5 | H1 | 1 | 50 | 54 | 2 | 61 | 64 | 3 | 124 | 130 | 4 | 141 | 145 | 5 | 212 | 217 | 6 | 409 | 416 | 7 | 468 | 470 |
| 5 | M2 | 1 | 46 | 50 | 2 | 57 | 60 | 3 | 120 | 126 | 4 | 137 | 141 | 5 | 208 | 213 | 6 | 381 | 388 | 7 | 440 | 442 |
| 5 | S1P1 | 1 | 68 | 72 | 2 | 79 | 82 | 3 | 141 | 147 | 4 | 157 | 161 | 5 | 223 | 228 | 6 | 276 | 283 | 7 | 338 | 340 |
