# Supplementary material for: Comparative Analysis of the Heptahelical Transmembrane Bundles of G Protein-Coupled Receptors
Source: PLoS One. 2012 Apr 24;7(4):e35802. doi: 10.1371/journal.pone.0035802 (PMC3335790; doi:10.1371/journal.pone.0035802)
Supplement: Table S3 — Sequence identity, similarity and rmsd among the representative chains of 10 receptors, for all 200 residues and for each of 5 sections. (DOC) [file pone.0035802.s006.doc]

Table S3: Sequence identity, similarity and rmsd among the representative chains of 10 receptors, for all 200 residues and for each of 5 sections.

| Name | brh | srh | β2 | β1 | A2A | CXCR4 | D3 | H1 | M2 |
| --- | --- | --- | --- | --- | --- | --- | --- | --- | --- |
| **all** |  |  |  |  |  |  |  |  |  |
| identity |  |  |  |  |  |  |  |  |  |
| brh | 100.0 |  |  |  |  |  |  |  |  |
| srh | 30.0 | 100.0 |  |  |  |  |  |  |  |
| β2 | 21.5 | 25.5 | 100.0 |  |  |  |  |  |  |
| β1 | 20.5 | 26.0 | 69.0 | 100.0 |  |  |  |  |  |
| A2A | 23.5 | 23.5 | 35.5 | 36.5 | 100.0 |  |  |  |  |
| CXCR4 | 22.0 | 21.5 | 24.0 | 24.0 | 22.0 | 100.0 |  |  |  |
| D3 | 28.0 | 20.5 | 38.0 | 41.5 | 32.5 | 27.5 | 100.0 |  |  |
| H1 | 20.0 | 23.0 | 36.5 | 37.0 | 34.0 | 25.0 | 35.5 | 100.0 |  |
| M2 | 22.5 | 20.5 | 29.5 | 31.0 | 27.0 | 23.0 | 33.0 | 37.5 | 100.0 |
| S1P1 | 21.5 | 23.5 | 27.0 | 28.5 | 29.5 | 22.0 | 26.5 | 24.5 | 27.0 |
| similarity |  |  |  |  |  |  |  |  |  |
| brh | 100.0 |  |  |  |  |  |  |  |  |
| srh | 63.5 | 100.0 |  |  |  |  |  |  |  |
| β2 | 49.0 | 50.0 | 100.0 |  |  |  |  |  |  |
| β1 | 48.0 | 50.5 | 87.0 | 100.0 |  |  |  |  |  |
| A2A | 53.0 | 50.5 | 56.5 | 59.0 | 100.0 |  |  |  |  |
| CXCR4 | 51.0 | 49.5 | 56.5 | 53.5 | 51.0 | 100.0 |  |  |  |
| D3 | 51.0 | 46.0 | 64.5 | 64.0 | 57.0 | 51.5 | 100.0 |  |  |
| H1 | 50.5 | 48.0 | 66.0 | 64.5 | 56.0 | 52.0 | 60.0 | 100.0 |  |
| M2 | 50.5 | 48.0 | 63.0 | 62.0 | 57.5 | 49.5 | 58.5 | 63.0 | 100.0 |
| S1P1 | 49.0 | 49.0 | 52.5 | 51.5 | 54.0 | 48.0 | 50.5 | 48.5 | 48.5 |
| rmsd |  |  |  |  |  |  |  |  |  |
| brh | 0.00 |  |  |  |  |  |  |  |  |
| srh | 1.66 | 0.00 |  |  |  |  |  |  |  |
| β2 | 1.91 | 1.94 | 0.00 |  |  |  |  |  |  |
| β1 | 1.95 | 2.03 | 0.55 | 0.00 |  |  |  |  |  |
| A2A | 2.42 | 2.41 | 2.05 | 2.06 | 0.00 |  |  |  |  |
| CXCR4 | 2.44 | 2.45 | 2.61 | 2.68 | 2.80 | 0.00 |  |  |  |
| D3 | 1.71 | 1.69 | 1.46 | 1.47 | 1.70 | 2.22 | 0.00 |  |  |
| H1 | 1.96 | 2.02 | 1.50 | 1.66 | 2.11 | 2.33 | 1.45 | 0.00 |  |
| M2 | 2.36 | 2.49 | 1.52 | 1.45 | 2.34 | 2.68 | 1.93 | 1.81 | 0.00 |
| S1P1 | 2.50 | 2.53 | 2.06 | 2.05 | 1.99 | 2.79 | 1.97 | 2.17 | 2.35 |
|  |  |  |  |  |  |  |  |  |  |
| **section1** |  |  |  |  |  |  |  |  |  |
| identity |  |  |  |  |  |  |  |  |  |
| brh | 100.0 |  |  |  |  |  |  |  |  |
| srh | 29.4 | 100.0 |  |  |  |  |  |  |  |
| β2 | 9.8 | 13.7 | 100.0 |  |  |  |  |  |  |
| β1 | 9.8 | 13.7 | 56.9 | 100.0 |  |  |  |  |  |
| A2A | 17.6 | 17.6 | 13.7 | 15.7 | 100.0 |  |  |  |  |
| CXCR4 | 11.8 | 9.8 | 7.8 | 9.8 | 9.8 | 100.0 |  |  |  |
| D3 | 21.6 | 11.8 | 17.6 | 27.5 | 9.8 | 13.7 | 100.0 |  |  |
| H1 | 15.7 | 7.8 | 17.6 | 15.7 | 15.7 | 17.6 | 17.6 | 100.0 |  |
| M2 | 13.7 | 11.8 | 11.8 | 19.6 | 5.9 | 15.7 | 15.7 | 23.5 | 100.0 |
| S1P1 | 11.8 | 15.7 | 13.7 | 19.6 | 11.8 | 5.9 | 9.8 | 7.8 | 9.8 |
| similarity |  |  |  |  |  |  |  |  |  |
| brh | 100.0 |  |  |  |  |  |  |  |  |
| srh | 49.0 | 100.0 |  |  |  |  |  |  |  |
| β2 | 31.4 | 39.2 | 100.0 |  |  |  |  |  |  |
| β1 | 37.3 | 41.2 | 82.4 | 100.0 |  |  |  |  |  |
| A2A | 43.1 | 39.2 | 29.4 | 33.3 | 100.0 |  |  |  |  |
| CXCR4 | 37.3 | 25.5 | 39.2 | 39.2 | 39.2 | 100.0 |  |  |  |
| D3 | 39.2 | 33.3 | 45.1 | 52.9 | 35.3 | 35.3 | 100.0 |  |  |
| H1 | 29.4 | 31.4 | 52.9 | 47.1 | 33.3 | 39.2 | 47.1 | 100.0 |  |
| M2 | 35.3 | 35.3 | 58.8 | 56.9 | 31.4 | 35.3 | 47.1 | 58.8 | 100.0 |
| S1P1 | 29.4 | 43.1 | 31.4 | 39.2 | 35.3 | 21.6 | 31.4 | 31.4 | 25.5 |
| rmsd |  |  |  |  |  |  |  |  |  |
| brh | 0.00 |  |  |  |  |  |  |  |  |
| srh | 1.97 | 0.00 |  |  |  |  |  |  |  |
| β2 | 2.45 | 2.79 | 0.00 |  |  |  |  |  |  |
| β1 | 2.55 | 2.96 | 0.70 | 0.00 |  |  |  |  |  |
| A2A | 3.59 | 3.82 | 3.36 | 3.35 | 0.00 |  |  |  |  |
| CXCR4 | 3.18 | 3.48 | 3.31 | 3.46 | 3.67 | 0.00 |  |  |  |
| D3 | 2.36 | 2.68 | 2.21 | 2.23 | 2.50 | 2.89 | 0.00 |  |  |
| H1 | 2.72 | 2.88 | 2.10 | 2.35 | 3.35 | 2.75 | 2.10 | 0.00 |  |
| M2 | 3.21 | 3.52 | 1.58 | 1.55 | 3.43 | 3.34 | 2.55 | 2.26 | 0.00 |
| S1P1 | 3.43 | 3.81 | 3.21 | 3.21 | 3.18 | 3.54 | 2.99 | 3.31 | 3.36 |
|  |  |  |  |  |  |  |  |  |  |
| **section2** |  |  |  |  |  |  |  |  |  |
| identity |  |  |  |  |  |  |  |  |  |
| brh | 100.0 |  |  |  |  |  |  |  |  |
| srh | 22.5 | 100.0 |  |  |  |  |  |  |  |
| β2 | 25.0 | 20.0 | 100.0 |  |  |  |  |  |  |
| β1 | 20.0 | 20.0 | 72.5 | 100.0 |  |  |  |  |  |
| A2A | 17.5 | 7.5 | 22.5 | 25.0 | 100.0 |  |  |  |  |
| CXCR4 | 25.0 | 25.0 | 22.5 | 15.0 | 17.5 | 100.0 |  |  |  |
| D3 | 25.0 | 7.5 | 42.5 | 50.0 | 27.5 | 17.5 | 100.0 |  |  |
| H1 | 10.0 | 22.5 | 47.5 | 47.5 | 30.0 | 22.5 | 37.5 | 100.0 |  |
| M2 | 15.0 | 12.5 | 22.5 | 25.0 | 22.5 | 12.5 | 27.5 | 40.0 | 100.0 |
| S1P1 | 17.5 | 10.0 | 22.5 | 17.5 | 25.0 | 27.5 | 12.5 | 27.5 | 27.5 |
| similarity |  |  |  |  |  |  |  |  |  |
| brh | 100.0 |  |  |  |  |  |  |  |  |
| srh | 55.0 | 100.0 |  |  |  |  |  |  |  |
| β2 | 45.0 | 42.5 | 100.0 |  |  |  |  |  |  |
| β1 | 47.5 | 47.5 | 90.0 | 100.0 |  |  |  |  |  |
| A2A | 47.5 | 37.5 | 47.5 | 55.0 | 100.0 |  |  |  |  |
| CXCR4 | 52.5 | 52.5 | 57.5 | 55.0 | 42.5 | 100.0 |  |  |  |
| D3 | 45.0 | 35.0 | 67.5 | 70.0 | 50.0 | 45.0 | 100.0 |  |  |
| H1 | 42.5 | 37.5 | 72.5 | 72.5 | 52.5 | 47.5 | 67.5 | 100.0 |  |
| M2 | 35.0 | 37.5 | 52.5 | 55.0 | 60.0 | 40.0 | 55.0 | 57.5 | 100.0 |
| S1P1 | 32.5 | 30.0 | 42.5 | 40.0 | 42.5 | 47.5 | 30.0 | 42.5 | 40.0 |
| rmsd |  |  |  |  |  |  |  |  |  |
| brh | 0.00 |  |  |  |  |  |  |  |  |
| srh | 1.53 | 0.00 |  |  |  |  |  |  |  |
| β2 | 1.74 | 1.48 | 0.00 |  |  |  |  |  |  |
| β1 | 1.86 | 1.61 | 0.53 | 0.00 |  |  |  |  |  |
| A2A | 1.70 | 1.51 | 1.33 | 1.50 | 0.00 |  |  |  |  |
| CXCR4 | 2.52 | 2.22 | 2.52 | 2.68 | 2.36 | 0.00 |  |  |  |
| D3 | 1.58 | 1.41 | 0.96 | 1.18 | 1.24 | 2.01 | 0.00 |  |  |
| H1 | 2.14 | 1.97 | 1.53 | 1.84 | 1.74 | 2.21 | 1.43 | 0.00 |  |
| M2 | 2.34 | 2.06 | 1.15 | 1.23 | 1.62 | 2.57 | 1.54 | 1.52 | 0.00 |
| S1P1 | 2.32 | 2.15 | 1.84 | 1.92 | 1.65 | 2.67 | 1.97 | 2.16 | 1.89 |
|  |  |  |  |  |  |  |  |  |  |
| **section3** |  |  |  |  |  |  |  |  |  |
| identity |  |  |  |  |  |  |  |  |  |
| brh | 100.0 |  |  |  |  |  |  |  |  |
| srh | 25.0 | 100.0 |  |  |  |  |  |  |  |
| β2 | 21.9 | 28.1 | 100.0 |  |  |  |  |  |  |
| β1 | 28.1 | 31.2 | 71.9 | 100.0 |  |  |  |  |  |
| A2A | 28.1 | 25.0 | 40.6 | 43.8 | 100.0 |  |  |  |  |
| CXCR4 | 40.6 | 21.9 | 31.3 | 28.1 | 25.0 | 100.0 |  |  |  |
| D3 | 37.5 | 21.9 | 56.3 | 53.1 | 37.5 | 53.1 | 100.0 |  |  |
| H1 | 25.0 | 28.1 | 43.8 | 46.9 | 43.8 | 37.5 | 59.4 | 100.0 |  |
| M2 | 28.1 | 25.0 | 43.8 | 43.8 | 37.5 | 43.8 | 50.0 | 65.6 | 100.0 |
| S1P1 | 18.8 | 25.0 | 37.5 | 43.8 | 43.8 | 28.1 | 40.6 | 40.6 | 43.8 |
| similarity |  |  |  |  |  |  |  |  |  |
| brh | 100.0 |  |  |  |  |  |  |  |  |
| srh | 68.8 | 100.0 |  |  |  |  |  |  |  |
| β2 | 59.4 | 50.0 | 100.0 |  |  |  |  |  |  |
| β1 | 53.1 | 50.0 | 84.4 | 100.0 |  |  |  |  |  |
| A2A | 65.6 | 59.4 | 71.9 | 75.0 | 100.0 |  |  |  |  |
| CXCR4 | 68.8 | 50.0 | 68.8 | 59.4 | 62.5 | 100.0 |  |  |  |
| D3 | 65.6 | 50.0 | 84.4 | 75.0 | 71.9 | 75.0 | 100.0 |  |  |
| H1 | 68.8 | 59.4 | 68.8 | 68.8 | 68.8 | 71.9 | 75.0 | 100.0 |  |
| M2 | 75.0 | 56.2 | 78.1 | 75.0 | 78.1 | 71.9 | 75.0 | 84.4 | 100.0 |
| S1P1 | 62.5 | 53.1 | 65.6 | 62.5 | 68.8 | 59.4 | 68.8 | 62.5 | 65.6 |
| rmsd |  |  |  |  |  |  |  |  |  |
| brh | 0.00 |  |  |  |  |  |  |  |  |
| srh | 1.38 | 0.00 |  |  |  |  |  |  |  |
| β2 | 1.51 | 1.16 | 0.00 |  |  |  |  |  |  |
| β1 | 1.44 | 1.25 | 0.37 | 0.00 |  |  |  |  |  |
| A2A | 1.79 | 1.56 | 0.95 | 1.10 | 0.00 |  |  |  |  |
| CXCR4 | 1.40 | 1.24 | 1.05 | 1.11 | 1.36 | 0.00 |  |  |  |
| D3 | 1.32 | 1.01 | 0.54 | 0.59 | 1.09 | 0.97 | 0.00 |  |  |
| H1 | 1.39 | 1.39 | 0.83 | 0.87 | 1.18 | 1.19 | 0.83 | 0.00 |  |
| M2 | 1.74 | 1.77 | 1.02 | 1.03 | 1.12 | 1.37 | 1.17 | 0.92 | 0.00 |
| S1P1 | 1.85 | 1.66 | 0.99 | 1.13 | 0.90 | 1.46 | 1.08 | 1.05 | 1.07 |
|  |  |  |  |  |  |  |  |  |  |
| **section4** |  |  |  |  |  |  |  |  |  |
| identity |  |  |  |  |  |  |  |  |  |
| brh | 100.0 |  |  |  |  |  |  |  |  |
| srh | 41.0 | 100.0 |  |  |  |  |  |  |  |
| β2 | 33.3 | 43.6 | 100.0 |  |  |  |  |  |  |
| β1 | 33.3 | 43.6 | 82.1 | 100.0 |  |  |  |  |  |
| A2A | 38.5 | 38.5 | 59.0 | 61.5 | 100.0 |  |  |  |  |
| CXCR4 | 28.2 | 35.9 | 33.3 | 33.3 | 30.8 | 100.0 |  |  |  |
| D3 | 33.3 | 38.5 | 46.2 | 43.6 | 56.4 | 33.3 | 100.0 |  |  |
| H1 | 28.2 | 28.2 | 46.2 | 41.0 | 43.6 | 28.2 | 33.3 | 100.0 |  |
| M2 | 28.2 | 35.9 | 41.0 | 38.5 | 46.2 | 28.2 | 46.2 | 30.8 | 100.0 |
| S1P1 | 43.6 | 41.0 | 41.0 | 41.0 | 46.2 | 30.8 | 51.3 | 30.8 | 41.0 |
| similarity |  |  |  |  |  |  |  |  |  |
| brh | 100.0 |  |  |  |  |  |  |  |  |
| srh | 79.5 | 100.0 |  |  |  |  |  |  |  |
| β2 | 69.2 | 76.9 | 100.0 |  |  |  |  |  |  |
| β1 | 66.7 | 74.4 | 89.7 | 100.0 |  |  |  |  |  |
| A2A | 66.7 | 71.8 | 76.9 | 74.4 | 100.0 |  |  |  |  |
| CXCR4 | 61.5 | 84.6 | 64.1 | 59.0 | 59.0 | 100.0 |  |  |  |
| D3 | 61.5 | 66.7 | 76.9 | 74.4 | 76.9 | 59.0 | 100.0 |  |  |
| H1 | 69.2 | 66.7 | 79.5 | 71.8 | 69.2 | 61.5 | 59.0 | 100.0 |  |
| M2 | 66.7 | 66.7 | 66.7 | 69.2 | 66.7 | 59.0 | 69.2 | 61.5 | 100.0 |
| S1P1 | 76.9 | 71.8 | 76.9 | 76.9 | 76.9 | 66.7 | 74.4 | 69.2 | 74.4 |
| rmsd |  |  |  |  |  |  |  |  |  |
| brh | 0.00 |  |  |  |  |  |  |  |  |
| srh | 1.43 | 0.00 |  |  |  |  |  |  |  |
| β2 | 1.61 | 1.25 | 0.00 |  |  |  |  |  |  |
| β1 | 1.58 | 1.22 | 0.36 | 0.00 |  |  |  |  |  |
| A2A | 1.81 | 1.33 | 0.94 | 0.94 | 0.00 |  |  |  |  |
| CXCR4 | 1.51 | 1.33 | 1.81 | 1.80 | 2.02 | 0.00 |  |  |  |
| D3 | 1.29 | 0.85 | 0.81 | 0.74 | 0.98 | 1.44 | 0.00 |  |  |
| H1 | 1.28 | 1.10 | 0.85 | 0.86 | 1.17 | 1.60 | 0.79 | 0.00 |  |
| M2 | 1.21 | 1.44 | 1.17 | 1.06 | 1.40 | 1.73 | 1.06 | 0.97 | 0.00 |
| S1P1 | 1.79 | 1.44 | 1.01 | 0.99 | 0.71 | 2.02 | 0.97 | 1.09 | 1.35 |
|  |  |  |  |  |  |  |  |  |  |
| **section5** |  |  |  |  |  |  |  |  |  |
| identity |  |  |  |  |  |  |  |  |  |
| brh | 100.0 |  |  |  |  |  |  |  |  |
| srh | 31.6 | 100.0 |  |  |  |  |  |  |  |
| β2 | 21.1 | 26.3 | 100.0 |  |  |  |  |  |  |
| β1 | 15.8 | 26.3 | 65.8 | 100.0 |  |  |  |  |  |
| A2A | 18.4 | 31.6 | 50.0 | 44.7 | 100.0 |  |  |  |  |
| CXCR4 | 10.5 | 18.4 | 31.6 | 39.5 | 31.6 | 100.0 |  |  |  |
| D3 | 26.3 | 26.3 | 36.8 | 39.5 | 39.5 | 28.9 | 100.0 |  |  |
| H1 | 23.7 | 34.2 | 34.2 | 42.1 | 44.7 | 23.7 | 39.5 | 100.0 |  |
| M2 | 31.6 | 21.1 | 36.8 | 34.2 | 31.6 | 21.1 | 34.2 | 36.8 | 100.0 |
| S1P1 | 18.4 | 28.9 | 26.3 | 26.3 | 28.9 | 23.7 | 26.3 | 23.7 | 21.1 |
| similarity |  |  |  |  |  |  |  |  |  |
| brh | 100.0 |  |  |  |  |  |  |  |  |
| srh | 71.1 | 100.0 |  |  |  |  |  |  |  |
| β2 | 47.4 | 44.7 | 100.0 |  |  |  |  |  |  |
| β1 | 39.5 | 42.1 | 89.5 | 100.0 |  |  |  |  |  |
| A2A | 47.4 | 50.0 | 68.4 | 68.4 | 100.0 |  |  |  |  |
| CXCR4 | 42.1 | 42.1 | 60.5 | 60.5 | 57.9 | 100.0 |  |  |  |
| D3 | 50.0 | 50.0 | 57.9 | 52.6 | 60.5 | 52.6 | 100.0 |  |  |
| H1 | 52.6 | 52.6 | 60.5 | 68.4 | 65.8 | 47.4 | 57.9 | 100.0 |  |
| M2 | 50.0 | 50.0 | 63.2 | 57.9 | 63.2 | 50.0 | 52.6 | 57.9 | 100.0 |
| S1P1 | 52.6 | 50.0 | 55.3 | 44.7 | 55.3 | 55.3 | 57.9 | 44.7 | 47.4 |
| rmsd |  |  |  |  |  |  |  |  |  |
| brh | 0.00 |  |  |  |  |  |  |  |  |
| srh | 1.79 | 0.00 |  |  |  |  |  |  |  |
| β2 | 1.82 | 2.07 | 0.00 |  |  |  |  |  |  |
| β1 | 1.84 | 2.07 | 0.61 | 0.00 |  |  |  |  |  |
| A2A | 2.12 | 2.16 | 1.84 | 1.75 | 0.00 |  |  |  |  |
| CXCR4 | 2.66 | 2.65 | 3.17 | 3.16 | 3.39 | 0.00 |  |  |  |
| D3 | 1.47 | 1.30 | 1.65 | 1.54 | 1.81 | 2.73 | 0.00 |  |  |
| H1 | 1.49 | 1.83 | 1.45 | 1.44 | 1.59 | 3.09 | 1.30 | 0.00 |  |
| M2 | 2.39 | 2.60 | 2.26 | 2.05 | 2.69 | 3.27 | 2.48 | 2.52 | 0.00 |
| S1P1 | 2.28 | 2.22 | 1.74 | 1.54 | 1.80 | 3.30 | 1.52 | 1.75 | 2.69 |
